# Targeting the Hippo Pathway in Gastric Cancer and Other Malignancies in the Digestive System: From Bench to Bedside

**DOI:** 10.3390/biomedicines10102512

**Published:** 2022-10-08

**Authors:** Xiaoli Liu, Yifei Wang, Bonan Chen, Wai Nok Chan, Chun Wai Mui, Alvin H.K. Cheung, Jinglin Zhang, Kit Yee Wong, Jun Yu, Wei Kang, Ka Fai To

**Affiliations:** 1 State Key Laboratory of Translational Oncology, Department of Anatomical and Cellular Pathology, Prince of Wales Hospital, The Chinese University of Hong Kong, Hong Kong 999077, China; 2 State Key Laboratory of Digestive Disease, Institute of Digestive Disease, The Chinese University of Hong Kong, Hong Kong 999077, China; 3 Sir Y.K. Pao Cancer Center, Li Ka Shing Institute of Health Science, The Chinese University of Hong Kong, Hong Kong 999077, China; 4 CUHK-Shenzhen Research Institute, The Chinese University of Hong Kong, Shenzhen 518000, China; 5 Department of Medicine and Therapeutics, The Chinese University of Hong Kong, Hong Kong 999077, China

**Keywords:** Hippo pathway, YAP1 (yes-associated protein 1), TAZ (WW domain-containing transcription regulator 1), gastric cancer

## Abstract

The Hippo pathway is an evolutionally conserved signaling cascade that controls organ size and tissue regeneration under physiological conditions, and its aberrations have been well studied to promote tumor initiation and progression. Dysregulation of the Hippo tumor suppressor signaling frequently occurs in gastric cancer (GC) and other solid tumors and contributes to cancer development through modulating multiple aspects, including cell proliferation, survival, metastasis, and oncotherapy resistance. In the clinic, Hippo components also possess diagnostic and prognostic values for cancer patients. Considering its crucial role in driving tumorigenesis, targeting the Hippo pathway may greatly benefit developing novel cancer therapies. This review summarizes the current research progress regarding the core components and regulation of the Hippo pathway, as well as the mechanism and functional roles of their dysregulation in gastrointestinal malignancies, especially in GC, and discusses the therapeutic potential of targeting the Hippo pathway against cancers.

## 1. Introduction

Gastric cancer (GC) is an aggressive malignant disease that arises from the stomach and represents the fourth leading cause of cancer-associated deaths worldwide [1]. Although treatment of GC, such as adjuvant chemo- or radiotherapies, has been dramatically improved, most GC patients are diagnosed at advanced stages with a worse prognosis and limited therapeutic options. Therefore, gaining new insights into the cancer biology of GC is essential for identifying effectors that drive gastric carcinogenesis and developing new therapeutic strategies against GC.

The Hippo pathway is highly conserved signaling throughout Drosophila melanogaster to mammals and acts as a master controller of organ size and tissue regeneration [2,3,4,5]. In the past decade, increasing evidence has emerged to support the contribution of the dysfunctional Hippo pathway, especially core Hippo signaling effectors yes-associated protein 1 (YAP1) and WW domain-containing transcription regulator 1 (TAZ), in tumor initiation, progression, and recurrence. In addition, aberrant expression or activation of the Hippo pathway is frequently observed in various cancer types, including GC, and exhibits excellent clinical value in diagnosis and prognosis for monitoring disease status. Thus, understanding the underlying mechanism by which the Hippo signaling augments tumor-facilitating properties can help to develop promising approaches for patients suffering from cancers.

Accumulative research has delineated the molecular mechanisms of Hippo-YAP1/TAZ signaling in GC, and some research has focused on developing novel therapeutic strategies targeting Hippo pathway-related cross talks. It is necessary to summarize the current research progress of the Hippo pathway in GC and other gastroenterology (GI) malignancies. In this review, we will introduce the main kinase cascade of the Hippo pathway, the aberrant activation of Hippo-YAP1/TAZ signaling in gastric carcinogenesis, the recent findings of the Hippo pathway in other GI cancers; and the clinical significance of Hippo signatures in GI cancers. With the deep investigation of the Hippo pathway in tumorigenesis, kinds of therapeutic strategies were developed for targeting the upstream or downstream of this pathway. This review will also update the identification of small molecule inhibitors for explicitly targeting the oncogenic components in the Hippo pathway. Finally, we will summarize the recent findings and point out our concerns and future directions. With further investigation of the Hippo pathway by cutting-edge techniques in GI cancer, we believe we can develop more precise strategies for eliminating Hippo-deregulated cancer.

## 2. Kinase Cascade of the Hippo Pathway

In mammalian cells, the canonical axis of the Hippo pathway comprises core kinases, including mammalian Ste20-like kinases 1/2 (MST1/2), salvador homolog 1 (SAV1), large tumor suppressor1/2 (LATS1/2), and MOB kinase activator 1 (MOB1) [6], as well as two transcriptional co-activators, YAP1 and TAZ. As critical downstream effectors of the Hippo pathway, YAP1 and TAZ lack DNA-binding motifs and thus function through interacting with TEA domain transcription factors (TEADs) to regulate the expression of target genes responsible for tumor cell proliferation and survival.

**Figure 1:**
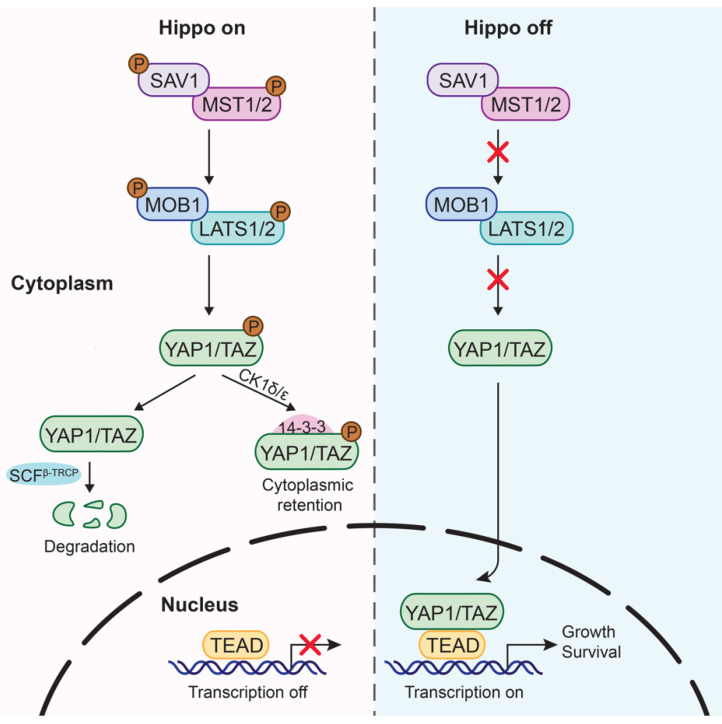
Core components of the Hippo pathway in human cells. When the Hippo pathway is ON, MST1/2 and SAV1 in complex phosphorylate LATS1/2 and MOB1/2. Activated LATS1/2 then phosphorylate YAP1 and TAZ, leading to YAP1/TAZ cytoplasmic retention or ubiquitin-mediated proteasomal degradation. On the other hand, when the Hippo pathway is OFF, dephosphorylated YAP1/TAZ translocate into the nucleus, where they bind with TEADs to induce the transcription of downstream target genes involved in cell growth and survival.

In general, activation of the Hippo pathway involves a series of phosphorylation cascades (Figure 1). MST1/2 works in complex with SAV1 to phosphorylate and activate LATS1/2 and its regulatory partner MOB1, which further induces the phosphorylation of YAP1 and TAZ for their inactivation. Phosphorylated YAP1 and TAZ then lead to 14-3-3 protein-dependent YAP1/TAZ cytoplasmic retention or ubiquitylation-induced degradation [7,8]. By contrast, inactivated upstream Hippo kinases and their partners prevent YAP1 and TAZ from phosphorylation, facilitating their nuclear translocation and subsequent transcription [9,10,11]. Nuclear localized YAP1 and TAZ could form the YAP1/TAZ-TEAD complex by binding with transcriptional factors TEAD1-4 to modulate subsequent gene transcription [12,13]. Taken together, Hippo effectors YAP1 and TAZ are tightly controlled by upstream kinases and regulators, and they exhibit an active form when they accumulate in the nuclei, while cytoplasmic sequestration leads to an inhibitory effect. Various genes responsible for cell growth and survival have been illustrated as direct targets of YAP1 and TAZ, and many of them are also involved in tumorigenesis upon dysregulation.

In the past years, accumulating evidence has focused on the causal relationship between irregular activation of the Hippo pathway, particularly YAP1 and TAZ, and tumor occurrence in numerous cancer types. Upon dysregulation, YAP1 and TAZ have also been illustrated to possess the tumor-facilitating potentials that contribute to tumor initiation and progression [14,15,16,17,18].

## 3. Hippo Pathway and GC

Mounting evidence proves that the Hippo pathway plays an important role in the initiation, progression, and recurrence of GC. In GC cell lines and human GC tumors, there is a consistent downregulation of Hippo kinases, including MST1/2 and LATS1/2, and an upregulation of downstream YAP1 and TAZ. Moreover, YAP1 signatures are preferentially expressed in the microsatellite stability/epithelial-to-mesenchymal transition (MSS/EMT) subtype group of GC [16]. The expression pattern of these Hippo components was closely associated with clinicopathologic features and prognosis of GC patients and could serve as reliable diagnostic and prognostic biomarkers in the clinic.

### 3.1. Functional Roles of Dysregulated Hippo Signaling in GC

Numerous studies suggest that dysregulated Hippo components coordinate to promote GC malignancy for tumor initiation, progression, and recurrence. As the most common tumor features, uncontrolled cell proliferation and survival have been well studied to be attributed to aberrant Hippo signaling inside GC tumors [19,20]. GC cells with lower MST1/2 or LATS1/2, concordant with high YAP1- and TAZ-expressing ones, possessed higher cell proliferative capability [21,22]. Upstream MST1 exerted its tumor-suppressive role in GC through a mechanism by which MST1 hindered the AMPK-Sirt3 pathway to cause excessive mitochondrial fission for cell death [23]. YAP1 and TAZ sustained enhanced cell proliferation mainly through a mechanism involving the transcriptional regulation of genes associated with cell division and cell cycle progression [24]. Targeting YAP1 resulted in impaired viability and colony formation in human GC cell lines and suppressed tumor growth in carcinogen N-methyl-N′-nitro-N-nitrosoguanidine (MNNG)-induced and *Helicobacter pylori* (*H. pylori*)-associated GC models as well [25]. Except for YAP1/TAZ, the upregulation of their well-established downstream targets, such as CTGF, BIRC5, and AREG, has also been revealed to drive gastric carcinogenesis and tumor progression [26,27]. Furthermore, dysfunctional Hippo signaling contributes to the enhanced metastatic capability of GC. Ectopically expressed YAP1 in GC MKN45 cells without YAP1 expression potentiated the malignant transition to a more invasive phenotype [28], whereas TAZ knockdown attenuated the ability of GC cells to migrate and invade [29]. In diffuse-type GC, activation of the YAP1 signaling induced by KRT17-mediated cytoskeletal reorganization elevated the IL-6 level, further accelerating EMT for tumor metastasis [30].

In addition, YAP1 was responsible for the stem-like properties of GC. In peritoneal carcinomatosis cells derived from GC patients, highly expressed YAP1 was positively associated with stemness-related signatures, such as SOX9, HES1, CD133, ITGA6, and ALDH1 [31]. High YAP1-expressing peritoneal carcinomatosis cell subset was also prone to develop PDX tumors, while genetic knockout or pharmacological inhibition of YAP1 abolished its tumorigenicity [31]. Furthermore, TAZ directly interacted with TEAD4 to augment the TAZ-dependent formation of vasculogenic mimicry and EMT in GC cells [32]. However, it is worth noting that despite YAP1 and TAZ having functional overlaps, integrative transcriptome analysis on GC cells identified the more dominant role of YAP1 in cell-substrate junction [33].

Taken together, dysregulation of the Hippo pathway promotes GC by regulating sustained proliferative signaling, enhanced metastasis, resistance to cell death, cancer stemness, and tumoral vascular formation.

### 3.2. The Upstream Regulatory Mechanisms on Hippo Signaling in GC

In the upstream, several regulators target different Hippo components. FBXW5 inactivated the Hippo pathway by enhancing LATS1 ubiquitination and degradation, and thus facilitated the metastasis and drug resistance of GC cells [34]. WBP2 inhibited LATS2 phosphorylation through direct interaction with the N-terminus kinase domain of LATS2 [35]. It further increased YAP-TEAD transcriptional activity and subsequently enhanced cell migration in GC [35]. Tang et al. demonstrated that STRN3 recruited MST1/2 to protein phosphatase 2A (PP2A) core enzyme complex for MST1/2 dephosphorylation, resulting in YAP1 hyperactivation [36]. Leukaemia inhibitory factor (LIF) activated tumor-suppressive LATS1/2 to diminish gastric cancer stemness cells (CSCs) and enhance the cancer-killing effect of 5-FU and doxorubicin [37]. Apart from the classical Hippo regulation, YAP1 can also be modulated by a non-canonical pathway. Protease-activated receptor-1 (PAR1) stimulated YAP1 activation via Rho, which further caused GC cell migration and stem-like features [38]. IRF3 enhanced the YAP1-TEAD4 complex interaction to increase YAP1 activity, whereas the blockade of IRF3 by amlexanox abolished GC growth [39]. The interaction between AMOTL1 and YAP1 protected YAP1 from ubiquitin-mediated degradation and facilitated nuclear translocation, thereby enhancing the tumorigenic role of YAP1 in GC [40]. In contrast, tumor suppressor RUNX3 was directly associated with TEAD via the Runt domain, which covered the DNA-recognition helix of TEAD and thus blocked its binding with DNA in GC [41]. As a result, the ability of the TEAD-YAP1 complex to promote GC cell proliferation was also hindered [41]. Furthermore, MST4–YAP axis has been demonstrated to be indispensable for GC development. Tumor suppressor MST4 kinase induced YAP1 phosphorylation at Thr83 to impede its interaction with importin α, thus restricting YAP1 activation. Depleting MST4 diminished YAP1 hyperactivation and thereby promoted GC growth [42]. Netrin-1 upregulated YAP1 via its classical receptor neogenin to promote GC cell metastasis as knockdown of neogenin abolished Netrin-1-induced YAP1 phosphorylation [43].

In addition, lncRNAs (e.g., RP11-323N12.5, LINC00649, and LncRNA MYLK-AS1) and microRNAs (e.g., microRNA-15a, microRNA-141, and microRNA-506) were also involved in Hippo dysregulation in GC [44,45,46,47,48,49,50]. LncRNAs MYLK-AS1 and LATS2-AS1-001 exerted their tumor-suppressive role by binding with EZH2 to increase LATS2 activity [44,50]. RP11-323N12.5 induced YAP1 transcription by binding to the c-MYC on its promoter region, resulting in enhanced GC progression and immunosuppression [45], while LINC00649 facilitated YAP1-dependent gastric tumorigenesis via sponging miR-16-5p [46]. For microRNAs, elevated miR-424 caused by circLARP4 repressed LATS1 translation to promote GC [51]. On the contrary, microRNA-141 directly downregulated TAZ for GC tumor suppression [47].

**Figure 2:**
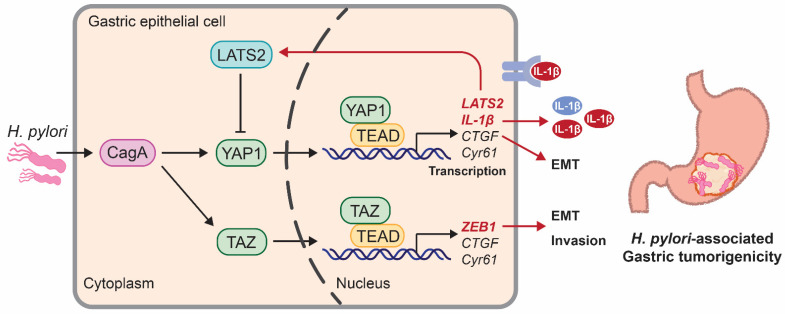
The role of *H. pylori* in Hippo pathway remodeling. CagA-positive *H. pylori* infection activates YAP1 and TAZ. CagA promoted the nuclear YAP1 and TAZ levels inside gastric mucosa from gastritis, and intestinal metaplasia, to GC. The Hippo signature genes, such as CTGF, Cyr61, IL-1β (Interleukin-1β), LATS2, etc., are upregulated following *H. pylori* infection. Moreover, TAZ participates in *H. pylori*-driven EMT and gastric carcinogenesis. In the gastric mucosa of *H. pylori*-associated GC mouse models and patients, nuclear TAZ accumulation activated ZEB1 expression, potentiating the EMT and stemness.

Recently, Hippo signaling has been illustrated to control the development of *H. pylori*-driven GC [52,53,54,55] (Figure 2). Chronic infection of *H. pylori,* especially those cytotoxin-associated gene A (CagA) positive strains, is a major risk factor for GC as it commonly delivers the oncoprotein CagA into gastric epithelial cells for tumorigenesis. The gradual increase in LATS2 and nuclear YAP1 levels was observed inside infected gastric mucosa from gastritis, and intestinal metaplasia, to GC [52]. Other Hippo signature genes, such as MST1, MST1R, vestigial-like family member 4 (VGLL4), and CTGF, were also upregulated following *H. pylori* infection [52]. Mechanistically, Molina-Castro et al. reported that LATS2 abolished *H. pylori*-induced EMT and the intestinal metaplasia phenotype, thereby preventing the occurrence of gastric tumors [52]. In addition, gastric epithelial cells infected with CagA-positive *H. pylori* have been shown to activate YAP1 and thus promote EMT and tumor growth of GC [53]. CagA-positive *H. pylori* stimulated YAP1 nuclear translocation and transactivated the proinflammatory IL-1ß for GC cell proliferation [54]. Furthermore, TAZ participated in *H. pylori*-driven EMT and gastric carcinogenesis [55]. Increased nuclear TAZ expression was observed in the gastric mucosa of *H. pylori*-associated GC mouse models and patients, which further upregulated EMT marker zinc finger E-box-binding homeobox 1 (ZEB1) to potentiate the EMT and stemness induced by *H. pylori* infection [55]. In addition to *H. pylori*, viruses also induced YAP1 hyperactivation, as RNA-seq analyses revealed an enrichment of YAP1 signature genes in human GC HGC-27 cells infected with the Sendai virus strain [39].

Furthermore, posttranslational modifications also affected the Hippo activity. N6-methyladenosine modification of YAP1 by METTL3 facilitated GC growth and migration [56]. The extracellular matrix stiffness has recently been identified to determine the hypermethylation of the YAP1 promoter [57]. The activated YAP1 further upregulated DNA methylation inhibitors, including GRHL2, TET2, and KMT2A to counteract the methylation for enhanced tumorigenic properties of GC under a stiff matrix [57].

Taken together, *H. pylori* promote the activation of YAP1/TAZ in gastric carcinogenesis, or the aberrant inaction of upstream quenches the suppressive effects of Hippo on YAP1/TAZ, leading to the YAP1/TAZ nuclear accumulation and oncogenic transformation. Then, how does YAP1/TAZ drive gastric carcinogenesis?

### 3.3. Downstream Effectors of Hippo Pathway in GC and Crosstalks between Signalings

In the downstream effectors, MYC is a direct downstream effector of YAP1/TAZ that has been identified to initiate GC [16]. In a mice model with conditional LATS1/2 knockout (*Lats1/2^iΔLgr5^*), specifically activated YAP1 and TAZ in Lgr5^+^ epithelial stem cells induced GC initiation via transcriptional upregulation of MYC, whereas inhibition of MYC significantly attenuated the YAP1/TAZ-driven GC development both in vitro and in vivo [16]. Transactivation of SLC35B4 induced by the YAP1-TEAD complex also led to GC progression [58]. Moreover, YAP1 is bound to peroxisome proliferator-activated receptors (PPARs) for SOX9 transactivation. It further caused enhanced tumorigenic function of GC [59]. Qiao et al. demonstrated that overexpression of YAP1 potentiated GC tumor metastasis through modulating cytoskeletal arrangement [60]. YAP1 induced RhoA suppressor ARHGAP29 transcription to promote actin depolymerization by turning F-actin into G-actin, thus causing enhanced metastatic capability of GC [60]. In addition, YAP1 was reported to potentiate GC cell survival, migration, and invasion by inhibiting endoplasmic reticulum stress via ERK signaling [61].

The main regulatory mechanisms for the Hippo pathway and the direct YAP1 downstream targets in gastric carcinogenesis have been summarized in Table 1.

### 3.4. Therapeutic Potential of Targeting the Hippo Signaling in GC

To date, chemotherapy has acted as the standard treatment for advanced GC; however, the acquired resistance has become a major clinical challenge since it vastly limits the therapeutic efficiency [63,64,65,66]. Recent studies have reported that YAP knockdown enhanced the sensitivity of GC cells to cisplatin, indicating that targeting YAP may improve the chemotherapeutic efficacy of GC [67].

Enhanced stability and nuclear accumulation of YAP1 induced by EphA2 mediated the 5-fluorouracil (5-FU) resistance in GC both in vitro and in vivo [68]. Furthermore, annexin A6 in cancer-associated fibroblast extracellular vesicles (CAF-EV) conferred cisplatin-resistant phenotypes in GC cells by activating the FAK-YAP1 signaling via stabilizing β1 integrin at the cell surface [69]. Pharmacological inhibition of YAP1 could effectively reverse the CAF-EV-induced drug resistance [69]. Trastuzumab is the only FDA-approved drug for the treatment of HER2-positive GC. YAP1 was highly expressed within trastuzumab-resistant GC tumors, where YAP1 activated by HER4 promoted EMT and subsequent trastuzumab resistance [70]. However, the detailed mechanism behind YAP-driven resistance warrants further investigation.

## 4. Hippo Pathway and Other Digestive Malignancies

In addition to GC, other malignancies in the digestive system (also known as GI cancer), such as colorectal, liver, esophageal, and pancreatic cancers, also threaten millions of patients’ lives [71]. Similar to GC, dysregulated Hippo signatures are commonly found in these GI cancers and have been proved to drive their malignant features. In this section, we summarized the main mechanisms of Hippo signaling pathway and its core components in other GI malignancies, apart from GC (Table 2).

**Colorectal cancer (CRC):** MST1/2 and LATS1/2 were downregulated in CRC, while the overexpressed YAP1 and TAZ were predominantly localized in the nucleus and consistently elevated in human CRCs from multiple cohorts [72]. In particular, increased YAP1 expression was positively associated with CRC patients’ clinicopathological characteristics, such as nodal status and TNM stage [86]. Previous studies have revealed that overexpressed YAP1 in human CRC cells was mainly attributed to dysregulated Wnt/β-catenin signaling through a mechanism by which β-catenin and TCF4 in a complex bound onto the DNA enhancer of YAP1 to induce its transactivation [78].

LATS1 functions as a tumor suppressor to diminish CRC growth and migration via glioma-associated oncogene homolog 1 (Gli1) downregulation [87]. Phosphorylation of MST1 and LATS2 promoted by metallothionein 2A (MT2A) restrained the YAP1 nuclear localization, thereby preventing CRC from enhanced tumor growth and liver metastasis [88]. In most CRC cases, upregulated YAP/TAZ commonly exerts oncogenic functions. Knockdown YAP or TAZ inhibited the ability of CRC cells to proliferate, form colonies, migrate, and invade, while co-silencing YAP and TAZ displayed synergistic inhibitory effects [77]. Mechanistically, YAP mainly drives CRC proliferation by activating the β-catenin signaling, thereby promoting the expansion of the colon CSCs population [75]. The increase in YAP enables *KRAS*-dependent CRC cell survival under KRAS suppression, as evidenced by ectopic expression of YAP1 in *KRAS*-mutated CRC cell lines could effectively restore cell viability [89]. YAP1-mediated transcription program of EMT is also responsible for potentiating the carcinogenetic effect of *KRAS* [89]. In addition, the Lats2/Sav1/YAP1/HIF1A signaling modulated by miR-103a-3p also controlled the glycolysis within CRC tumors through modulating glycolytic enzymes (e.g., HK2, PFK1, and LDHA) [90]. However, it is worth noting that aberrant YAP1 and TAZ have recently been considered tumor suppressors in CRC in several studies [91,92,93,94].

**Liver cancer:** Hepatocellular carcinoma (HCC) and intrahepatic cholangiocarcinoma (ICC) are the most common primary liver cancers that account for the majority of all cases [95]. The nuclear abundance of YAP1 was commonly observed in human HCC samples [7], possibly due to the amplification of YAP1 on 11q22 amplicon [96]. Zhou et al. demonstrated that since Mst1/2 are canonical YAP regulators, depletion of Mst1/2 abolished the phosphorylation of YAP and thus promoted YAP-driven liver enlargement and hepatocarcinogenesis [73]. RNA-binding motif protein 3 (RBM3) induced YAP1 upregulation and potentiated the cell proliferation of HCC [80]. Ubiquitin-specific peptidase 10 (USP10) was reported to interact with YAP1 and TAZ to suppress their ubiquitination, resulting in accelerated HCC tumor growth [97]. By contrast, the YAP1 activity suppressed by miR-375 or miR-590-5P led to impaired cell proliferation and invasion [81,82]. Furthermore, as we know, hepatitis B virus (HBV) infection is a critical driver of HCC initiation. It has been demonstrated that HBV X protein (HBx) transcriptionally upregulated YAP1 expression in a cAMP-response element binding protein (CREB)-dependent manner [98]. Consistently, hepatocytes expressing HBV preΔS2 mutation caused ER dysfunction and YAP1 activation, leading to inflammation, fibrosis, hepatomegaly, and eventually hepatocarcinogenesis in vivo [99].

In addition to HCC, ubiquitous activation of TAZ was observed in human ICC, another subtype of primary liver cancer. Using a mice model with TAZ and AKT coactivation (AKT/TAZ mice), Cigliano et al. identified that TAZ^S89A^ and AKT collaboratively contributed to ICC through a mechanism by which TAZ interacted with TEAD to induce the transdifferentiation of mature hepatocytes. At the same time, the blockade of the TAZ-TEAD binding merely led to an enrichment of lipid-rich giant cells [100]. Moreover, the knockdown of YAP1 also delayed the formation of AKT/TAZ-initiated ICC [100].

Translationally, YAP1 and TAZ are also involved in sorafenib resistance, the first-line targeted drug for advanced HCC [101,102]. YAP1/TAZ coordinated with ATF4 to transactivate SLC7A11, which further conferred HCC resistance to sorafenib [101]. Moreover, this process could also be driven by YAP1-mediated survival expression [102]. Inhibition of the Hippo-YAP1 signaling using verteporfin (VP) in combination with transcatheter arterial chemoembolization (TACE), a commonly used therapeutic approach for unresectable HCC, allows reduced tumor burden and improved survival in a transplanted HCC mice model [103].

**Esophageal cancer (EC):** EC cell lines and human EC samples often express constitutively active YAP1 and TAZ [104], which is significantly correlated with poor survival and adverse clinicopathological features such as histological classification and tumor stage [105]. Four YAP1 isoforms (α, ß, γ, δ) were detected in human ESCC cell lines, all of which are essential for cell proliferation [105]. Depletion of YAP1 suppressed cell proliferation, migration, and invasion but enhanced cell apoptosis in vitro and inhibited tumor growth in vivo [83,104]. YAP1 and Gli1 have been demonstrated to form a positive feedback loop to promote EC development, where Gli1 upregulated YAP1 by suppressing LATS1-mediated phosphorylation, which increased Gli1 level through direct regulation of PI3K/AKT pathway [83]. Moreover, TAZ displayed similar clinical significance and oncogenic functions as YAP1 did in EC [106,107] and was responsible for tribbles pseudokinase 3 (TRIB3)-driven cancer stemness and radioresistance of ESCC [106,108].

**Pancreatic ductal adenocarcinoma (PDAC):** Several studies have reported that YAP1 and TAZ are overexpressed and hyperactivated in human PDACs, facilitating the proliferation and metastasis of PC cells. Knocking down the expression of YAP1 decreased cell growth and colony formation in PC cell lines [109]. YAP1 can also regulate the expression of genes associated with secreted factors, which collectively maintain the neoplastic proliferation and tumorigenic stromal responses of the tumor microenvironment [110]. Moreover, increased YAP1 promoted the EMT in PC cells via activation of the AKT pathway and conferred resistance to gemcitabine [111]. YAP1/TAZ have been demonstrated to be downstream effectors in the malignant transformation of both the KRAS pathway and the mitogen-activated protein kinase (MAPK) pathway. *KRAS* mutations occur in about 90% of PDAC patients and have been well studied to promote PDAC initiation in transgenic models by stimulating YAP1/TAZ to trigger JAK-STAT3 signaling activation [85]. Activation of JAK-STAT3 then modulated the inflammatory cytokines’ response and thus promoted the progression from pancreatic intraepithelial neoplasias (PanIN) to PDAC in KRas^G12D^ mice [85]. Overall, these studies suggest that targeting YAP1/TAZ might provide clinical benefits to patients with PC.

## 5. Clinical Significance of Hippo Signatures in GI Cancers

**Figure 3:**
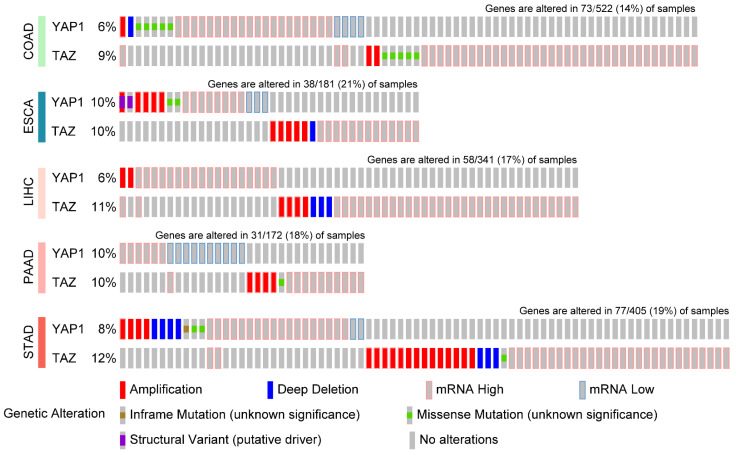
Genetic aberrations and mRNA upregulation of YAP1 and TAZ in gastrointestinal cancers. The TCGA PanCancer dataset illustrated the proportion of YAP1/TAZ alterations (including gene amplification, deep deletion, inframe and missense mutations, structural variants, and mRNA changes) in primary digestive cancers. Only positive cases with alterations were demonstrated in this Figure. COAD, colon adenocarcinoma; ESCA, esophageal carcinoma; LIHC, liver hepatocellular carcinoma; PAAD, pancreatic adenocarcinoma; STAD stomach adenocarcinoma.

**Figure 4:**
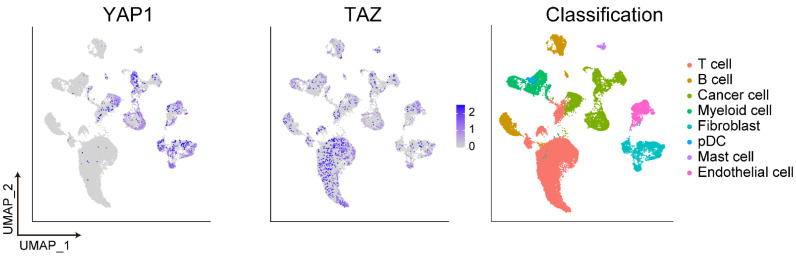
The expression of YAP1 and TAZ in the GC microenvironment. By scRNA-seq analysis, YAP1 was predominantly expressed in cancer cells, cancer-associated fibroblasts, and endothelial cells, while the immune cells (T and B cells) demonstrated less YAP1/TAZ expression. TAZ expression was diffusely distributed in the different cell types, with slightly higher expression in T cells.

To comprehensively characterize the genetic and epigenetic alterations of Hippo signature genes in GI cancers, we analyzed the genomics data of YAP1 and TAZ in different GI cancer types from the TCGA PanCancer database (Figure 3). For example, among the 405 GC patients, YAP1 and TAZ showed 8% and 12% of genetic alterations and mRNA upregulation, respectively. By single-cell RNA-seq analysis of data provided by Stanford Medicine [112], YAP1 was found predominantly expressed in cancer cells, fibroblasts, and endothelial cells, while TAZ expression was diffusely distributed with slightly higher expression in T cells [113] (Figure 4).

Clinically, YAP1 and TAZ can be used as diagnostic and prognostic tools values as their upregulation is not only responsible for early tumor detection but also predicts a worse prognosis in patients suffering from GI cancers [76,114]. In GC, there was a gradual increase of approximately three-fold in the ratio of YAP1-to-VGLL4 protein expression from GC grade I to grade IV, and their mRNA ratio was closely associated with aggressive tumor features [25]. For YAP1-positive GC, those with positive VGLL4 had significantly worse survival than those with negative VGLL4, suggesting that a combination of YAP1 and VGLL4 showed superior efficacy for GC surveillance [25]. TAZ alone is also capable of predicting tumor metastasis for patients diagnosed with gastric cardia adenocarcinoma [29]. In CRC, YAP1 and TAZ are independent prognostic factors associated with poor clinical outcomes [77,115]. Notably, CRC patients with high levels of YAP1 and TAZ experienced the comparatively shortest overall survival, suggesting that the combination of YAP1 and TAZ achieved the highest predictive value compared to YAP1 or TAZ alone [77]. In addition to YAP1/TAZ, serum MST1 level also aids early CRC detection, which showed high sensitivity and specificity of 92.3% and 100% when combined with carcinoembryonic antigen (CEA) and the fecal occult blood test (FOBT) [116].

In addition, YAP1 and TAZ could monitor therapy resistance, as elevated YAP1 expression was observed in 5-FU-resistant CRC and EC cell lines cells as well as in doxorubicin-resistant HCC HepG2 and Huh7 cells. The acquired resistance possibly arose from the YAP1/TAZ-induced EGFR upregulation [82,117,118,119]. In addition to chemoresistance, YAP1 and TAZ are also implicated in developing radiotherapy resistance [120,121,122]. Thus, YAP1 and TAZ may be utilized as an indicator of chemo- or radiotherapy response to select the optimal treatment for cancer patients.

In summary, the main Hippo components with aberrant expression in GI cancers can serve as prognostic or predictive biomarkers, especially indicating chemo- or radioresistance and tumor recurrence.

## 6. Therapeutic Strategies for Directly Targeting the Hippo Pathway

Given the crucial role of Hippo signaling in driving malignancies, many inhibitors targeting different Hippo components have been developed to provide new insights for cancer therapies.

**Verteporfin (VP):** Over 3300 compounds from the FDA-approved drug library underwent toxicological, pharmacokinetic, and pharmacodynamic tests in search of YAP1-specific inhibitors, where VP stood out as the initially discovered small molecule inhibitor [123,124]. VP has been demonstrated to inhibit YAP1/TAZ signaling and YAP1-potentiated oncogenic properties by disrupting the interaction between YAP1-TEAD in a transgenic murine model [124]. In GC, VP inhibited the expression of YAP1/TAZ-TEAD to suppress stem cell sphere formation and tumorigenesis [125]. Moreover, VP reduced the cyclinD1, MMP2, and Ang2 expression levels via blockade of the YAP1-TEAD complex, thus suppressing cell viability, angiogenesis, and angiogenic mimicry in PDAC [126]. In head and neck squamous carcinoma, VP downregulated the expression of EMT and CSC-associated genes through YAP1 in a light-dependent manner [127]. Strikingly, VP synergistically enhanced the antitumor effect of 5-FU in GC, as evidenced by significantly elevated cell apoptosis and reduced tumor growth in vivo [68].

**CA3:** Using the TEAD/YAP1 luciferase reporter assay, CA3 was recently initially identified to be a potent inhibitor of the YAP1-TEAD complex [128]. Functionally, CA3 suppressed the growth of YAP1^high^ esophageal adenocarcinoma cells, mesothelioma cancer, and oral squamous epithelial cell carcinoma [129,130]. In addition, CA3 treatment restrained cancer stemness and tumorigenicity of cells derived from GC peritoneal metastases in a YAP1-dependent mechanism [31].

**Statins:** The anticancer effect of statins has recently been proposed to act by targeting YAP1/TAZ. Mechanistically, statins such as cerivastatin and simvastatin induce YAP1/TAZ phosphorylation and inhibit their nuclear translocation via blockade HMG-CoA conversion to mevalonic acid, thereby affecting the expression of their target genes [131,132,133]. The inhibition of statins on YAP1/TAZ activity could be strongly reinforced when combined with dasatinib [134]. These statins could functionally inhibit the development of pancreatic intraepithelial neoplasia [131,135].

**Metformin:** An AMP-activated protein kinase (AMPK) activator that has recently been proved to target the YAP1-TEAD complex in solid tumors. Metformin induced YAP1 phosphorylation to abolish the YAP1-dependent stem-like properties in glioma [136]. In addition, metformin can serve as the sensitizer of 5-FU in HCC through modulating YAP1 [137].

**TEAD inhibitors:** VGLL4 is a transcriptional co-factor that could bind onto TEAD via its Tondu (TDU) domains [138,139]. Recent studies demonstrated that VGLL4 exhibited tumor-suppressive roles in many cancer types, a process mainly depends on its competition with YAP1 for binding to TEAD [140]. Moreover, a peptide mimicking VGLL4 (also known as Super-TDU) has been shown to decrease GC growth by targeting the interaction of YAP1-TEAD [25].

Since auto-palmitoylation of TEADs at a conserved cysteine is essential for maintaining their stabilization and functional roles [141,142], researchers have found that MGH-CP1 could directly target the auto-palmitoylation of TEAD to inhibit YAP1/TAZ-mediated transcriptional activation. Compounds 1 functions by binding to TEAD lipid pockets and damaging S-palmitoylation [143]. By evaluating the binding domain of YAP1 and TEADs, Pobbati et al. indicated that the YAP1-TEAD complex showed a significantly high affinity to a nonsteroidal anti-inflammatory drug, fluphenazine. TED-347 and its derivatives disrupt the protein interaction between TEAD-YAP1, thus inhibiting TEAD transcriptional activity [143,144,145,146,147]. A high-throughput drug screening identified VT101 and VT102 as selective TEAD palmitoylation inhibitors in NF2-deficient mesothelioma [148]. One recent study verified that K-975, a novel TEAD inhibitor, showed potent and high-selective inhibitory effects on the YAP1/TAZ-TEAD interaction in malignant pleural mesothelioma [149]. All these small molecules could inhibit YAP1-driven malignant properties by disrupting TEAD activity, suggesting their potential as candidates for promising cancer therapies.

**Other compounds potentially target the Hippo pathway for GC treatment:** Tyrosine kinase inhibitors, such as dasatinib and pazopanib, have been shown to elicit phosphorylation and degradation of YAP1/TAZ for their cytoplasmic sequestration [150]. The therapeutic efficacy of these tyrosine kinase inhibitors also relies on the expression and activity of YAP1/TAZ. In GC SNU-1 and SNU-484 cells, 3,3′-Diindolylmethane exerted the antitumor effect by promoting the binding of RASSF1 with the MST1/2-LATS1-Mob1 complex, which further led to Hippo activation and YAP1 phosphorylation [151].

As indicated in Table 3, we summarized some current clinical studies of selected Hippo pathway inhibitors in GI cancers, including GC (https://clinicaltrials.gov, accessed on 6 September 2022).

## 7. Conclusions

Investigations on the Hippo signaling pathway field have rapidly expanded since Kieran F. Harvey first discovered it nearly 20 years ago. With the evidence available now, the Hippo signaling is not only dysregulated in GC and other GI cancers but also confers multiple pro-tumorigenic traits such as sustaining cell proliferation, survival, and metastasis for tumor progression. YAP1/TAZ acts as a determining effector of the Hippo pathway to drive tumorigenesis. From the diagnosis and prognosis aspects, the decreased expression of Hippo kinases and increased YAP1 and TAZ within tumor tissues serve as indicators for clinical outcomes of GI cancer patients. In addition, tumoral Hippo components could provide intriguing tools for early detection and therapeutic strategies of cancers.

## 8. Discussion

The past research has focused on the molecular mechanisms of the Hippo pathway, especially on the mRNA/protein upregulation or signal transduction. Meanwhile, as prognostic or predictive biomarkers, the main Hippo components have been highlighted in GI cancer. However, the current knowledge is far from presenting the whole image of the Hippo pathway in GI cancers as more mechanisms and hallmarks have emerged, such as the microbiota in shaping the Hippo pathway, the Hippo pathway in regulating the tumor microenvironment, etc. Meanwhile, with the development of high technologies and concepts, such as single-cell RNA sequencing and tumor evolution, it is urgent to differentiate the aberrant activation of Hippo components in different cell types and how they communicate with each other to drive carcinogenesis. For small molecule inhibitor development, endeavors are required to identify more potent inhibitors with fewer side effects, as so far, no small molecule has been successfully approved for clinical use.

## 9. Future Directions

To successfully translate the current knowledge from basic studies into clinical practice, we need to address several questions: First, in addition to what is known, it is still unclear which transcription factors predominantly activate YAP1/TAZ transcriptionally to drive GC progression in the *H. pylori*-positive cases. Meanwhile, the downstream effectors of the Hippo pathway that facilitate GC cancer initiation and progression should be further identified and investigated. Second, characterizing the specific mechanism behind Hippo-mediated therapy resistance, with the aim of improving the efficacy of chemo- and radiotherapies, is urgently needed. In addition, the potential role of Hippo signaling in modulating other tumor-facilitating properties (e.g., metabolic reprogramming, immune escape, and maintaining CSC properties) of GC remains unclear. Third but not least, YAP1 and TAZ have been shown to exert opposite functions in specific cancer types pending further clarification. For example, in CRC, many studies have reported that YAP1 and TAZ function as oncoproteins, while growing evidence suggests overexpressed YAP1 and TAZ could prevent CRC development [91,92,93,94].

Taken together, the dysregulated Hippo pathway plays a pivotal role in GI cancer progression; serves as a diagnostic, predictive, and surveillance biomarker; and provides a potential therapeutic target for GI tumor eradication. A deep investigation of the Hippo pathway in GI malignancies will not only broaden our knowledge about tumor progression but also provide novel therapeutic strategies for eliminating cancer.

## Figures and Tables

**Table 1:** Summary of upstream regulators and downstream targets of the Hippo pathway in GC.

Regulators	Targets	Regulatory Mechanisms	References
FBXW5	LATS1	FBXW5 inactivates the Hippo pathway by enhancing LATS1 ubiquitination and degradation, which promotes GC cell invasion, metastasis, and drug resistance.	[34]
circLARP4		circLARP4 increases miR-424 level, which in turn inhibits LATS1 translation.	[51]
WBP2	LATS2	WBP2 directly interacts with LATS2 promoter to inhibit LATS2 and activate the YAP1-TEAD transcriptional activity.	[35]
lncRNAs		Both MYLK-AS1 and LATS2-AS1-001 bind with EZH2 to induce LATS2 activation.	[44,50]
LIF	LATS1/2	Activates LATS1/2 to suppress the malignant properties of CSCs.	[37]
PAR1	YAP1	PAR1 activates YAP1 in a Rho-dependent manner.	[38]
IRF3		IRF3 strengthens the interaction between YAP1 and TEAD4 for YAP1 activation.	[39]
AMOTL1		AMOTL1 associates with YAP1 to prevent the ubiquitin-mediated YAP1 degradation, thus promoting its nuclear translocation and subsequent tumorigenic roles.	[40]
MST4		MST4 phosphorylates YAP1 at Thr83 to impede its interaction with importin α for YAP1 inactivation.	[42]
Netrin-1		Netrin-1 phosphorylates YAP1 via its receptor neogenin.	[43]
RP11-323N12.5		LncRNA RP11-323N12.5 binds with c-MYC to transactivate YAP1.	[45]
METTL3		METTL3 regulates the m6A modification of YAP1 for its activation.	[56]
NUSAP1		NUSAP1 stabilizes YAP1 protein through direct binding.	[62]
KRT17		KRT17 loss stimulates RhoA-dependent F-actin polymerization and induces YAP1 signaling activation and nuclear localization.	[30]
microRNA-141	TAZ	microRNA-141 directly downregulates TAZ.	[47]
RUNX3	TEAD	RUNX3 interacts with TEAD to abrogate the DNA binding ability of TEAD, thus limiting the oncogenic property of the TEAD-YAP1 complex in GC.	[41]
*H. Pylori*	Hippo components	Controls the expression of MST1, LATS2, YAP1, and TAZ to promote EMT and other tumorigenic properties.	-
**Downstream Targets of YAP1 in GC**		**Regulatory Mechanisms**	**References**
YAP1	MYC	Upon YAP1 activation, activated YAP1 transcriptionally modulates MYC by binding to MYC enhancer and sequesters p72 to hinder miRNAs-mediated MYC suppression.	[16]
	IL-1ß	YAP1-TEAD complex binds onto IL-1ß promoter to transcriptionally upregulate IL-1ß production, thus resulting in GC cell proliferation.	[54]
	SLC35B4	YAP1-TEAD complex induces SLC35B4 transactivation via binding to the promoter region of SLC35B4.	[58]
	SOX9	YAP1 binds to peroxisome proliferator-activated receptors (PPARs) to induce transcriptional regulation of SOX9, which further leads to enhanced cancer stemness and tumorigenicity.	[31,59]
	ARHGAP29	YAP1 transcriptionally upregulates ARHGAP29 to suppress the RhoA-LIMK-cofilin pathway for F-actin destabilization. The YAP1-ARHGAP29 signaling is essential for GC metastasis.	[60]

**Table 2:** Summary of the main Hippo signaling pathway core components in GI malignancies.

Members	Cancer Type	Expression	Function	Mechanisms	Reference
MST1/2	CRC	Downregulated	Tumor suppressor	Depletion of Mst1/2 driven CRC.	[72]
	HCC			Mst1/2 kinases are canonical YAP/TAZ regulators.	[73]
LATS1/2	CRC	Downregulated	Tumor suppressor	LATS1/2 can inhibit CRC growth and metastasis via downregulating Gli1 expression and MT2A-mediated absence of YAP1 nuclear localization.	[72,74,75]
	HCC			Depletion of LAST1/2 lead to hyperactivation of YAP/TAZ.	[74]
YAP1/YAZ	CRC	Upregulated	Oncogene	The expression of YAP1 is driven via aberrant Wnt/β-catenin signaling in human CRC cells.	[75,76,77,78]
	HCC			YAP1/TAZ activates mTORC1 in HCC cells. The interaction of RBM3 -YAP1 is important for HCC proliferation, and USP10 was reported to inhibit YAP1/TAZ ubiquitinating. Overexpression of miR-375 and miR-590-5P inhibited cell proliferation and invasion by downregulating YAP1 activity.	[79,80,81,82]
	EC			Gli1 upregulated YAP1 with LATS1 independent mode. Simultaneously, YAP1 increased Gli1 expression by direct regulation PI3K/AKT pathway.	[83]
	PDAC			YAP1/TAZ acts as an essential downstream in the oncogenic transition of both KRAS and MAPK pathways. *KRAS*-mutation promotes PDAC initiation in transgenic models by activating YAP1/TAZ to upregulate JAK-STAT3 signaling activation.	[84,85]

**Table 3:** Clinical trials on Hippo inhibitors-based therapy in GI cancers.

ClinicalTrials.gov Identifier	Official Title	Condition or Disease	Intervention/Treatment	Phase	Status
NCT00944463	Trial of Simvastatin and Gemcitabine in pancreatic cancer patients	PDAC	Drug: Gemcitabine + simvastatin Drug: Gemcitabine+Placebo	2	Completed
NCT01813994	Role of Statin on gastric inflammation in patients at high risk of gastric cancer	Early GC or gastric adenoma	Drug: Arm1: Statin Drug: Arm2: Placebo	Not applicable	Completed
NCT02968810	Simvastatin in preventing liver cancer in patients with liver cirrhosis	Cirrhosis	Other: Placebo Administration Other: Questionnaire Administration Drug: Simvastatin	2	Active, not recruiting
NCT04947020	dataBase for analysis of rectal cancer oncological results (BARO)	Rectal cancer	Drug: Metformin	Not applicable	Recruiting
NCT01099085	Trial of XP (Capecitabine/CDDP) Simvastatin in advanced gastric cancer patients	GC	Drug: Simvastatin Drug: Placebo	3	Completed
NCT02569645	Rosuvastatin in the treatment of rectal cancer	Rectal cancer	Drug: Rosuvastatin	2	Completed
NCT01075555	Sorafenib Tosylate with or without pravastatin in treating patients with liver cancer and cirrhosis	Liver cancer	Drug: Pravastatin sodium Drug: Sorafenib tosylate	3	Completed
NCT02026583	A Single Arm, Phase II study of Simvastatin Plus XELOX and Bevacizumab as first-line chemotherapy in metastatic CRC Patients	CRC	Drug: Simvastatin	2	Completed
NCT01281761	Simvastatin + Cetuximab/Irinotecan in K-ras mutated CRC	Metastatic CRC	Drug: Cetuximab/irinotecan/simvastatin	2	Completed
NCT00313859	Phase II Study of Simvastatin Plus Irinotecan, Fluorouracil, and Leucovorin (FOLFIRI) for metastatic CRC	Metastasis CRC	Drug: Simvastatin	2	Completed
NCT03889795	Phase IB Metformin, Digoxin, Simvastatin in solid tumors	Advanced PDAC	Drug: Metformin Drug: Simvastatin Drug: Digoxin	1	Recruiting
NCT05368805	Fruquintinib DDI study with P-gp and BCRP substrates	Metastatic CRC	Drug: Fruquintinib Drug: Dabigatran Etexilate Drug: Rosuvastatin	1	Active, not recruiting
NCT04767984	Testing Atorvastatin to lower colon cancer Risk in longstanding ulcerative colitis	CRC and ulcerative colitis	Drug: Atorvastatin Calcium	2	Recruiting
NCT03033225	Ultrasound-Guided Verteporfin photodynamic therapy for the treatment of unresectable solid pancreatic tumors or advanced pancreatic cancer, VERTPAC-02 Study	Advanced PDAC Locally advanced PDAC Metastatic PDAC	Drug: Photodynamic Therapy Drug: Verteporfin	2	Recruiting

## Data Availability

All data are available within the manuscript.

## Abbreviations

5-FU, 5-fluorouracil; AMPK, AMP-activated protein kinase; CAF-EV, cancer-associated fibroblast extracellular vesicles; CagA, cytotoxin-associated gene A; CEA, carcinoembryonic antigen; CRC, colorectal cancer; CREB, cAMP-response element binding protein; CSCs, cancer stemness cells; EC, esophageal cancer; EMT, epithelial-to-mesenchymal transition; FOBT, fecal occult blood test; GC, gastric cancer; Gli1, glioma-associated oncogene homolog 1; HBV, hepatitis B virus; HBx, HBV X protein; *H. pylori*, Helicobacter pylori; HCC, hepatocellular carcinoma; HMG-CoA, hydroxy-3-methylglutaryl coenzyme A; ICC, intrahepatic cholangiocarcinoma; LATS1/2, large tumor suppressor1/2; LIF, leukaemia inhibitory factor; MAPK, mitogen-activated protein kinase; MNNG, *N-methyl-N′-nitro-N-nitrosoguanidine*; MOB1, MOB kinase activator 1; MSS, microsatellite stability; MST1/2, mammalian Ste20-like kinases 1/2; MT2A, metallothionein 2A; PanIN, pancreatic intraepithelial neoplasias; PAR1, protease-activated receptor 1; PDAC, pancreatic ductal adenocarcinoma; PP2A, protein phosphatase 2A; PPARs, peroxisome proliferator-activated receptors; RBM3, RNA binding motif protein 3; SAV1, salvador homolog 1; TACE, transcatheter arterial chemoembolization; TAZ, WW domain-containing transcription regulator 1; TDU, Tondu; TEADs, TEA domain transcription factors; TRIB3, tribbles pseudokinase 3; USP10, ubiquitin-specific peptidase 10; VGLL4, vestigial-like family member 4; VP, verteporfin; YAP1, yes-associated protein 1; ZEB1, zinc finger E-box-binding homeobox 1.
